# Characterisation of Physical Frailty and Associated Physical and Functional Impairments in Mild Cognitive Impairment

**DOI:** 10.3389/fmed.2017.00230

**Published:** 2017-12-18

**Authors:** Ma Shwe Zin Nyunt, Chang Yuan Soh, Qi Gao, Xinyi Gwee, Audrey S. L. Ling, Wee Shiong Lim, Tih Shih Lee, Philip L. K. Yap, Keng Bee Yap, Tze Pin Ng

**Affiliations:** ^1^Gerontology Research Programme, Department of Psychological Medicine, National University of Singapore, Singapore, Singapore; ^2^Department of Geriatric Medicine, Tan Tock Seng Hospital, Singapore, Singapore; ^3^Duke Medical School, National University of Singapore, Singapore, Singapore; ^4^Department of Geriatric Medicine, Khoo Teck Puat Hospital, Singapore, Singapore; ^5^Department of Geriatric Medicine, Ng Teng Fong General Hospital, Singapore, Singapore

**Keywords:** mild cognitive impairment, frailty, physical function, gait, strength

## Abstract

**Objective:**

To characterize the physical frailty phenotype and its associated physical and functional impairments in mild cognitive impairment (MCI).

**Method:**

Participants with MCI (*N* = 119), normal low cognition (NLC, *N* = 138), and normal high cognition (NHC, *N* = 1,681) in the Singapore Longitudinal Ageing Studies (SLAS-2) were compared on the prevalence of physical frailty, low lean body mass, weakness, slow gait, exhaustion and low physical activity, and POMA balance and gait impairment and fall risk.

**Results:**

There were significantly higher prevalence of frailty in MCI (18.5%), than in NLC (8.0%) and NHC (3.9%), and pre-frailty in MCI (54.6%), NLC (52.9%) than in NHC (48.0%). Age, sex, and ethnicity-adjusted OR (95% CI) of association with MCI (versus NHC) for frailty were 4.65 (2.40–9.04) and for pre-frailty, 1.67 (1.07–2.61). Similar significantly elevated prevalence and adjusted ORs of association with MCI were observed for frailty-associated physical and functional impairments. Further adjustment for education, marital status, living status, comorbidities, and GDS significantly reduced the OR estimates. However, the OR estimates remained elevated for frailty: 3.86 (1.83–8.17), low body mass: 1.70 (1.08–2.67), slow gait: 1.84 (1.17–2.89), impaired gait: 4.17 (1.98–8.81), and elevated fall risk 3.42 (1.22–9.53).

**Conclusion:**

Two-thirds of MCI were physically frail or pre-frail, most uniquely due to low lean muscle mass, slow gait speed, or balance and gait impairment. The close associations of frailty and physical and functional impairment with MCI have important implications for improving diagnostic acuity of MCI and targetting interventions among cognitively frail individuals to prevent dementia and disability.

## Introduction

Late life cognitive impairment and physical impairment are principal causes of disability, falls, hospitalisation, institutionalisation, and death among the elderly. In elderly persons, chronic disability rather than multi-morbidity is the strongest negative prognostic factor for functionality and survival (1), and in the oldest old, known predictors such as smoking and obesity lose their importance, whereas high disability level, poor physical, and cognitive performance, predict mortality (2). Based on accumulating evidence, it is increasingly appreciated that cognitive and physical impairment in late life are inter-related through shared pathophysiological mechanisms and could probably be manifestations of a single complex phenotype (3).

Mild cognitive impairment (MCI) and the physical frailty phenotype are early cognitive and physical syndromes preceding the development of dementia and disability among older people. MCI is a transitional state of cognition between normal ageing and dementia that may progress to dementia, remain stable, or reverse to normal cognition over a defined period of time. MCI is defined by subjective or objective evidence of cognitive decline greater than expected for the individual’s age and education level but that does not interfere notably with activities of daily life (4). Studies show that older individuals with MCI compared to their counterparts without cognitive impairment performed more poorly not just on tests of neurocognitive performance tasks, but also on tests of complex motor and psychomotor domains tasks (5–7), and exhibited greater gait impairment especially on tests that include motor-cognitive dual tasks (8–12). These motor functional deficiencies in MCI are also present in physical frailty, a syndrome that may also reverse to the robust state or progress to functional disability (13). The physical phenotype of frailty is represented by low levels of lean body mass, muscle strength, gait performance, physical activity (PA), and energy.

Studies suggest that gait and other physical manifestations of the frailty syndrome are associated with cognitive decline and dementia. For example, the presence of weight loss or being underweight is well known to precede the onset of Alzheimer’s disease (14), lower grip strength, and extremity motor performance were associated with cognitive decline and decreased risk of MCI, and MCI conversion to AD (7, 15), frailty was associated with incident AD and cognitive decline (14, 15), and low levels of PA was associated with cognitive decline (16).

Some authors have argued that motor functional changes should be considered clinical features of MCI, and complex psychomotor tests such as gait speed may be as useful as cognitive tests in the identification of MCI particularly among elderly patients with less education (17). Converging lines of research and consensus also advocate defining MCI more precisely in terms of cognitive-physical constructs of “cognitive frailty” (the simultaneous presence of both physical frailty and MCI) (18), or the analogous motoric cognitive risk (MCR) syndrome (presence of cognitive complaints and slow gait) (19). Few studies have described the prevalence of frailty and its physical and functional impairments in MCI. The associations of specific physical and functional impairments of frailty with MCI are also unclear.

In this population-based study of community dwelling, older persons in the second Singapore Longitudinal Ageing Study (SLAS-2), we compared the prevalence of the physical frailty syndrome, low lean muscle mass, low muscle strength, slow gait speed, exhaustion, low PA, impaired balance, impaired gait, and elevated fall risk between individuals with MCI and non-MCI individuals with normal (high and low) cognitive functioning. We also examined the effects of psycho-social and health-related factors on these associations. We hypothesise that the prevalence of the physical frailty syndrome and its physical and functional impairments are higher in MCI compared to their cognitively normal counterparts, and this association is independent of psycho-social and health-related factors.

## Materials and Methods

### Participants

This study was conducted as part of the Singapore Longitudinal Ageing Studies (SLAS), an ongoing prospective cohort observational study of community-dwelling older adults, aged 55 and above. A first wave (SLAS-1) cohort was recruited between 2003 and 2004 from the South-East region of Singapore. A second wave (SLAS-2) cohort was recruited between 2009 and 2011 in the South West and South-Central regions of Singapore. Participants were identified by door-to-door census, which had demographic characteristics similar to the rest of the population. Residents who were severely physically or mentally ill and incapacitated to give informed consent or participate were excluded. The study was approved by the National University of Singapore Institutional Review Board, and all participants signed written informed consent. Detailed descriptions of the methodology in the SLAS cohorts have been previously described (20).

In this study, we used baseline data from the participants of SLAS-2. A total of 3,270 older adults were enrolled at baseline with an estimated response rate of 78%. Trained research nurses and psychologists conducted questionnaire interviews, testing, and assessment to collect an extensive range of sociodemographic and health-related data. These included questionnaire and physical testing of frailty status, and multi-phasic cognitive screening, assessment and diagnosis of neurocognitive disorders. The participants included 2,844 Chinese, 259 Malay, 148 Indian, and 15 other ethnicities. After excluding participants who did not participate in neurocognitive tests, and those with dementia, there were 2,052 participants who were identified as MCI or normal cognition. Among them, 114 did not have complete frailty data. The final study sample thus comprised 1,938 subjects for analysis.

### Identification of MCI and Normal Cognition

The participants’ cognitive status was determined using a two-stage screening and diagnostic assessment process. In the first stage, global cognitive assessments were performed using the MMSE (21), and the Montreal Cognitive Assessment (MoCA) (22), which has been previously validated for use in the multiethnic population of Singapore in English, Malay, and Chinese languages (23, 24). Participants who were screened positive by scoring 26 or below on either the MMSE or MoCA underwent the Clinical Dementia Rating (CDR) assessment conducted by trained research nurses, and a comprehensive battery of neurocognitive testing conducted by psychology-trained research assistants, prior to consensus diagnosis of MCI (and dementia) or normal cognition by a panel of geriatricians and psychiatrists, who reviewed all relevant interview, testing, and assessment data.

The neurocognitive assessment included tests of memory [Rey Auditory Verbal Learning Test (25) and Story Memory (26)]; attention [longest span of the digit span subtest, forwards and backwards, from WAIS-III (27)]; visuospatial ability [Brief Visuospatial Memory Test-Revised (28) and Clock Reading Test (29)]; language [Boston Naming Test (30)]; executive functioning [Colour Trails Test 1 and 2 (31)]; and the Block Design subtest from the WAIS-III (27).

At a screening interview, a total of 1,681 participants who scored 27 and above on the MMSE and MOCA were denoted as normal (high) cognition (NHC) (32). There were a total of 138 participants who were screened positive on the MMSE or MOCA but were not assessed (*N* = 60) or provided incomplete or unreliable responses (*N* = 23) on the neurocognitive testing or the CDR, or did not meet the criteria for diagnosis of MCI or dementia (*N* = 55). These participants who were not successfully adjudicated as cases of MCI (or dementia) were assigned the status of normal low cognition (NLC). In 119 participants, MCI was defined according to criteria recommended by the MCI Working Group of the European Consortium on Alzheimer’s disease (33):

1. Personal or informant report of cognitive decline relative to previous abilities during the past year.
2. Objective deficit in one or more cognitive domains; defined as a score that was 1.5 SD below age and education adjusted norms (34).
3. CDR of 0.5 or Sum of Boxes score less than 3 (35).
4. Functional independence on basic activities of daily living (Barthel Index).
5. No dementia.

### Frailty and Physical Function Measures

*Physical frailty* was assessed by scores (1 = present, 0 = absent) for five components (shrinking, weakness, slowness, exhaustion, and low PA) proposed by Fried et al. (36) and used in the Cardiovascular Health Study (CHS), with the following operational modifications:

(i) Shrinking was defined by unintentional weight loss of 4 kg or more in the past 6 months, or a body mass index of less than 18.5 kg/m^2^, or calf circumference of 31 cm or less.
(ii) Weakness was assessed using knee extension strength measured using dominant knee extension, using the average value from three trials in kilograms, standardised on gender and BMI strata.
(iii) Slowness was assessed by the 6-m fast gait speed test using the average of two measurements, and the lowest quintile values stratified for gender and height to classify participants as slow, based on data in a previous large population-based study (17).
(iv) Exhaustion was measured as a combined score of three questions from the SF-12 quality of life scale, “Did you have lots of energy?” “Did you feel tired?” (reverse-scored) and, “Did you feel worn out?” (reverse-scored) (37). A score of <10 was used to denote exhaustion.
(v) Low PA was determined by the total amount of time spent on performing moderate and vigorous activities per week based on questions in the LASA PA questionnaire (38) that fell below the gender-specific lowest quintile determined in the forerunner SLAS-1 study.

As per the CHS criteria, participants were categorised by their total scores as robust (score = 0), pre-frail (score = 1–2), and frail (score = 3–5).

*Falls risk* was assessed using the Tinetti performance-oriented mobility assessment (POMA) (39). Balance was assessed using standard scoring criteria (0, 1, or 2) to grade sitting balance, standing balance immediately after arising, turning around, and other manoeuvres (total score 0–16). Gait performance (gait initiation, step length and height, symmetry, continuity, path deviation, trunk sway, and walking stance) by having the subject stand with examiner, walks down hallway or across the room, first at “usual” pace, then back at “rapid, but safe” pace (using usual walking aids), total score (0–12). Falls risk was assessed by total balance and gait scores of <19 = high fall risk, 19–24 = medium fall risk, and 25–28 = low fall risk.

*Covariates* measured included (i) sociodemographic data including age, gender, and education, living status (live alone), (ii) medical comorbidity (determined from self-reports of a known diagnosis and/or treatment of 14 specific conditions (hypertension, diabetes, high cholesterol, stroke, heart attack, atrial fibrillation, heart failure, cataracts, kidney failure, asthma, chronic obstructive pulmonary disease, arthritis, hip fracture), and/or other chronic conditions in the past year, and the total number of medical illnesses), (iii) lifestyle including current smoking and daily alcohol drinking, (iv) depressive symptoms [assessed by the Geriatric Depression Scale (GDS) (40)], (v) disability status assessed by dependency on basic activity of daily living (BADL) (41) and instrumental activities of daily living (42).

### Statistical Analyses

The prevalence of frailty and pre-frailty, low lean body mass, weakness, slow gait, exhaustion, low PA, impaired balance, impaired gait, and elevated fall risk were compared between MCI, NLC, and NHC using chi-squared tests of significance, and odds ratio and 95% confidence intervals (95% CI) of association estimated from logistic regression, adjusted for age, sex, and ethnicity. Further adjustment for education, marital status, living status, comorbidities, GDS, and IADL ability were performed to assess the effects of common psycho-social and health-related factors in mediating these associations.

## Results

The study participants comprised 119 (6.3%) MCI, 138 (7.2%) NLC (MMSE and MOCA scores <27), and 1,681 (85.5%) NHC (MMSE and MOCA scores ≥27) (Table 1). Among MCI participants, 18.5% were frail, compared to 8.0% among NLC and 3.9% among NHC. The prevalence of pre-frailty was similarly higher in MCI (54.6%) and in NLC (52.9%) than in NHC (48.0%) (Table 2). Age, sex, and ethnicity-adjusted OR (95% CI) of association with MCI (versus NHC) for frailty was 4.65 (2.40–9.04) and for pre-frailty was 1.67 (1.07–2.61). In addition, significantly higher prevalence of low lean body mass, weakness, slow gait, exhaustion, low PA, impaired balance, impaired gait, and elevated falls risk were observed in the MCI group than in the NLC and NHC groups (Table 2). The age, sex, and ethnicity-adjusted ORs of association with MCI ranged between 1.71 and 6.99 for these factors (Table 3).

**Table 1:** Demographic and personal characteristics among mild cognitive impairment (MCI), normal high cognition (NHC), normal low cognition (NLC) groups.

Variables (*N* = 1,938)	NHC^a^		NLC^b^		MCI		*P*-value*
Sample *N*		1,681		138		119	
Age; 55–64	38.8	(653)	23.2	(32)	31.9	(38)	<0.001
65–74	53.4	(897)	55.8	(77)	46.2	(55)	
≥75	7.8	(131)	21.0	(29)	21.9	(26)	
Male gender	37.8	(636)	33.3	(46)	36.1	(43)	0.55
Chinese ethnicity	91.1	(1,530)	90.6	(125)	80.7	(96)	0.001
Single, divorced, widow	27.4	(460)	57.2	(59)	49.6	(60)	<0.001
Education: none	8.1	(136)	38.4	(53)	43.7	(52)	<0.001
Primary	41.4	(694)	44.9	(62)	44.5	(53)	
Living status: alone	13.7	(229)	27.5	(38)	19.7	(23)	<0.001
Alcohol: yes	3.4	(57)	2.9	(4)	2.5	(3)	0.85
Smoking: non smoker	80.9	(1,360)	71.7	(99)	75.6	(90)	0.07
Ex-smoker	10.4	(175)	16.7	(23)	14.3	(17)	
Current smoker	8.7	(146)	11.6	(16)	10.1	(12)	
APOE-e4 allele	17.5	(264)	24.8	(29)	15.0	(18)	
MMSE	28.97	±1.14	25.85	±2.98	24.59	±3.50	<0.001
MoCA	27.7	±1.26	21.37	±3.74	19.57	±4.64	<0.001
GDS	0.51	±1.04	1.20	±2.15	1.10	±1.89	<0.001
Noof comorbidities	2.16	±1.41	2.61	±1.47	3.1	±1.71	<0.001
IADL disability	5.9	(98)	11.0	(15)	25.2	(30)	<0.001

*^a^NHC have MMSE and MOCA scores ≥27*.

*^b^NLC have MMSE and/or MOCA <27, but have no MCI diagnosis*.

*Figures are % (*N*) and mean ± SD*.

*P-values of significance are derived from Chi square test for categorical variables and ANOVA for continuous variables*.

**Table 2:** Physical frailty characteristics among mild cognitive impairment (MCI), normal high cognition (NHC), normal low cognition (NLC) groups.

Variables (*N* = 1,938)	NHC		NLC		MCI		P*
Sample *N*		1,681		138		119	
**Frailty status (global)**							
Robust	48.1	(809)	39.1	(54)	26.9	(32)	<0.001
Pre-frail	48.0	(807)	52.9	(73)	54.6	(65)	
Frail	3.9	(65)	8.0	(11)	18.5	(22)	
**Frailty status (domains)**							
Shrinking	18.5	(310)	28.3	(39)	33.6	(40)	<0.001
Slowness	15.0	(252)	21.7	(30)	37.8	(45)	<0.001
Weakness	11.3	(190)	16.7	(23)	24.4	(29)	<0.001
Exhaustion	14.3	(241)	12.3	(17)	23.5	(28)	0.017
Low physical activity (PA)	13.1	(220)	16.7	(23)	23.5	(28)	0.004
Impaired balance (POMA ≤14)	2.9	(48)	6.5	(9)	10.4	(12)	<0.001
Impaired gait (POMA ≤10)	2.3	(38)	6.5	(9)	14.3	(17)	<0.001
Medium-high fall risk (POMA ≤24)	1.1	(19)	3.6	(5)	8.7	(10)	<0.001
**Physical function measures**							
Frailty scores	0.74	±0.86	0.98	±0.99	1.43	±1.19	<0.001
Calf circumference	34.41	±3.72	33.67	±4.03	34.02	±5.83	0.07
BMI, kg/m^2^	59.89	±10.81	57.16	±10.47	59.45	±12.44	0.018
Gait speed, s	4.66	±1.49	5.25	±1.81	6.14	±2.77	<0.001
Knee extension, kg	16.40	±6.22	14.14	±6.23	12.93	±4.51	<0.001
Energy score	12.06	±2.19	11.94	±2.10	11.54	±2.34	0.041
PA level	11.53	±1.91	10.97	±1.48	10.85	±1.41	<0.001
POMA Balance score	15.86	±0.61	15.77	±0.19	15.68	±0.86	0.004
POMA Gait score	11.87	±0.74	11.70	±0.98	11.41	±1.42	<0.001
POMA total score	27.73	±1.13	27.47	±1.50	27.10	±1.88	<0.001

*Figures are % (*N*) and mean ± SD*.

*P-values of significance are derived from Chi square test for categorical variables and ANOVA for continuous variables*.

**Table 3:** Odds ratio of association of physical frailty status and components with cognitive status [mild cognitive impairment (MCI), normal low cognition (NLC), normal high cognition (NHC)].

		Unadjusted			Adjusted: age, sex, ethnicities			Adjusted: age, gender, ethnicity, education, APOE-e4, marital status, living status, comorbidities, GDS, IADL		
		OR	95% CI	*P*	OR	95% CI	*P*	OR	95% CI	*P*
Frailty versus robust	NHC	1			1			1		
	NLC	2.54	1.26–5.08	0.009	1.47	0.70–3.08	0.31	1.28	0.50–3.26	0.602
	MCI	8.56	4.70–15.57	<0.001	4.65	2.40–9.04	<0.001	3.49	1.47–8.31	0.005

Pre-frail versus robust	NHC	1			1			1		
	NLC	1.36	0.94–1.95	0.103	1.67	0.80–1.70	0.42	1.02	0.67–1.56	0.929
	MCI	2.04	1.32–3.14	0.001	1.67	1.07–2.61	0.02	1.37	0.85–2.23	0.197

Low body mass (Calf circumference ≤31)	NHC	1			1			1		
	NLC	1.74	1.18–2.57	0.005	1.37	0.91–2.06	0.13	1.28	0.81–2.04	0.294
	MCI	2.24	1.50–3.34	<0.001	1.74	1.14–2.64	0.01	1.58	0.96–2.59	0.071

Low muscle strength	NHC	1			1			1		
	NLC	1.57	1.0–2.52	0.06	1.21	0.74–1.96	0.45	1.10	0.63–1.92	0.747
	MCI	2.53	1.62–3.95	<0.001	1.79	1.12–2.86	0.02	1.65	0.95–2.87	0.075

Slow gait speed	NHC	1			1			1		
	NLC	1.58	1.03–2.41	0.04	1.19	0.76–1.85	0.45	0.89	0.53–1.49	0.667
	MCI	3.45	2.33–5.11	<0.001	2.36	1.56–3.58	<0.001	1.68	1.02–2.79	0.042

Impaired gait (POMA) (POMA ≤10)	NHC	1			1			1		
	NLC	2.35	1.13–4.90	0.02	2.78	1.28–6.02	0.01	1.97	0.78–4.95	0.148
	MCI	3.93	2.03–7.63	<0.001	5.53	2.87–10.65	<0.001	3.71	1.62–8.46	0.002

Impaired balance POMA (POA ≤16)	NHC	1			1			1		
	NLC	3.00	1.42–6.34	0.004	1.84	0.86–3.96	0.12	1.36	0.55–3.35	0.507
	MCI	7.17	3.91–13.14	<0.001	2.75	1.35–5.59	0.01	1.90	0.79–4.53	0.149

Medium-high fall risk (POMA ≤ 24)	NHC	1			1			1		
	NLC	3.25	1.20–8.84	0.02	3.17	1.13–8.91	0.03	1.55	0.40–6.03	0.528
	MCI	8.24	3.74–18.16	<0.001	6.99	2.96–16.51	<0.001	3.02	0.95–9.53	0.060

Exhaustion	NHC	1			1			1		
	NLC	0.84	0.50–1.42	0.52	0.81	0.47–1.38	0.43	0.51	0.26–0.98	0.042
	MCI	1.84	1.18–2.87	0.007	1.73	1.09–2.75	0.02	1.21	0.69–2.12	0.499

Low PA	NHC	1			1			1		
	NLC	1.33	0.83–2.12	0.24	1.14	0.70–1.84	0.60	1.03	0.60–1.77	0.921
	MCI	2.04	1.31–3.19	0.002	1.71	1.07–2.73	0.02	1.29	0.74–2.23	0.371

*Adjusted for age, sex, ethnicity, education, APOE-e4, marital status, living status, comorbidities, GDS, and IADL ability*.

To determine the effects of psycho-social and health-related factors influencing the observed association, further adjustment for education, marital status, living status, comorbidities, GDS, and IADL ability were performed and found to significantly reduce the OR of association (Table 3). However, the OR (95% CI) of association with MCI (versus NHC) remained significantly elevated for frailty: 3.86 (1.83–8.17); low body mass: 1.70 (1.08–2.67); slow gait: 1.84 (1.17–2.89); impaired gait: 4.17 (1.98–8.81); and elevated falls risk: 3.42 (1.22–9.53).

## Discussion

This study supports the strong and intimate relationship between cognitive and physical impairment, which are present in both MCI and physical frailty. The relationship may be explained by common underlying pathophysiological factors, which include pathways involved in the development of cardiovascular and cerebrovascular diseases, insulin-mediated metabolic disturbances, protein-calorie undernutrition, sex steroids, growth hormones, vitamin D, chronic inflammation, and oxidative stress (3).

In this study, almost two-thirds of community dwelling older adults with MCI manifested the physical syndrome of frailty or pre-frailty, including low lean muscle mass, low muscle strength, slow gait speed, exhaustion and low PA, as well as balance and gait impairment, which pose elevated risk of falls, in greater proportions compared to their cognitively normal counterparts. Psycho-social and health-related factors did not wholly account for the association, such that frailty, low lean body mass, slow gait speed, gait impairment, and impaired gait and balance measure of elevated falls risk remained independently associated with MCI. The OR estimates suggest a very strong association and appears to be specific for phenotypic measures of low lean body mass, slow gait speed, and gait and balance impairment, but not exhaustion or low PA.

Prior studies have shown that older persons with MCI exhibited greater gait variability especially during dual-tasking walking than cognitively normal controls (8, 9). Walking is a complex activity that involves executive functioning, spatial orientation, navigation, and memory, among other cognitive functions (43). The use of simple measures of gait speed or POMA balance and gait scores may thus complement cognitive tests in the identification of MCI among elderly patients especially those with less education (44). At least one other study have shown that the combination of cognitive complaints and slow gait (MCR syndrome) successfully predict increased risk of cognitive decline and dementia (19). However, it remains unclear which components or combinations of physical frailty and cognitive impairment are most optimal in identifying cognitively frail older persons.

In the years, since the conceptual definition of MCI was first proposed, numerous studies have shown that non-cognitive manifestations such as depressive and neuropsychiatric symptoms (45–47), sensory impairment such as in hearing (48), or smell (49), and subtle IADL impairments involving complex functions (50, 51) are over-represented in MCI significantly more than non-MCI controls, and were able to enhance the ability of MCI to predict future risks of dementia. This is also true of physical functional impairment that co-occurs in MCI. Cases of MCI with concomitant physical frailty may be considered to fulfil the criteria for cognitive frailty (18). Taken together, these findings suggest that the understanding of MCI beyond the conceptual confines of cognitive impairment may help to improve diagnostic acuity and present meaningful targets for interventions among cognitively frail individuals to prevent dementia and disability.

In this regard, the cognitive frailty concept has potential clinical and research advantages in better stratifying the risk profiles of older people for developing dementia and functional disability. Recent studies have shown that the cognitive frailty construct more accurately predict greater risks of cognitive decline and dementia than MCI alone (15, 44, 52, 53). However, it has not been determined whether it is also in fact a more stable construct than MCI, in being less liable to revert to cognitive normal. Another point to note is the prevalence of the cognitive frailty construct. In this study, the prevalence is very low (1.1%) if cases were defined by 22 frail MCI subjects (out of 1,938 participants), but is higher (4.5%) if cases were defined by 65 pre-frail plus 22 frail MCI subjects. It is possible that in this study, the overly restrictive criteria used to define both the cognitive and physical components of this construct may contribute to under-estimating its prevalence, as further discussed below.

The diagnosis of MCI in this study was based on clinical panel consensus review of relevant data according to internationally recommended criteria and is a strength of this study. However, the restrictive criteria for diagnosis of MCI may exclude subjects akin to cases labelled in some studies as “cognitive impairment-no dementia” (CIND). Doubtful cases of MCI were consigned into the category of NLC, a heterogeneous group of subjects, which also included those with below normal global performance on the MMSE or MOCA but who failed to provide supportive cognitive domain or CDR data to merit a MCI diagnosis or otherwise. On close scrutiny, this NLC group appeared to include significantly more participants who were living alone and with higher GDS depression scores, a possible explanation for their failed clinical assessment. The results for NLC showed a pattern of relationship with frailty and its associated physical and functional impairments that was intermediate between cognitive (high) normal and MCI, but with no significantly strong associations with physical functional impairments.

The results for frailty components of exhaustion (fatigue) and low PA were negative. However, this may reflect the limitations of our operationalised measurement of these phenotypic features, and further studies using more sensitive and discriminating instruments are required to ascertain the replicability of these findings. Because of the small numbers, we did not further distinguish MCI participants into amnestic or non-amnestic subtypes. Further studies should investigate the ability of combined cognitive, physical, and functional markers of MCI in predicting future risks of developing dementia.

## Ethics Statement

This study was carried out in accordance with the recommendations of Human Biomedical Research Act, Singapore Ministry of Health; with written informed consent from all subjects. All subjects gave written informed consent in accordance with the Declaration of Helsinki. The protocol was approved by the National University of Singapore IRB.

## Role of the Sponsors

The sponsors had no role in the conduct of the study or preparation of this manuscript.

## Author Contributions

TN formulated the hypothesis, designed the study, reviewed the data, and revised the manuscript. WL, TL, PY, and KY reviewed clinical data and adjudicated on MCI diagnosis. GQ performed the data preparation. MN analysed the data. CYS reviewed the literature, drafted and revised the manuscript; all authors participated in the study design and data collection, reviewed the results and manuscript, and approved the manuscript submission.

## Conflict of Interest Statement

The authors declare that the research was conducted in the absence of any commercial or financial relationships that could be construed as a potential conflict of interest.

## References

[1] Marengoni A von Strauss E Rizzuto D Winblad B Fratiglioni L The impact of chronic multimorbidity and disability on functional decline and survival in elderly persons. A community-based, longitudinal study. J Intern Med (2009) 265(2):288–95. 10.1111/j.1365-2796.2008.02017.x 19192038

[2] Nybo H Petersen HC Gaist D Jeune B Andersen K McGue M Predictors of mortality in 2,249 nonagenarians – the Danish 1905-cohort survey. J Am Geriatr Soc (2003) 51(10):1365–73. 10.1046/j.1532-5415.2003.51453.x 14511155

[3] Panza F Seripa D Solfrizzi V Tortelli R Greco A Pilotto A Targeting cognitive frailty: clinical and neurobiological roadmap for a single complex phenotype. J Alzheimers Dis (2015) 47(4):793–813. 10.3233/JAD-150358 26401761

[4] Gauthier S Reisberg B Zaudig M Petersen RC Ritchie K Broich K Mild cognitive impairment. Lancet (2006) 367(9518):1262–70. 10.1016/S0140-6736(06)68542-5 16631882

[5] Kluger A Gianutsos JG Golomb J Ferris SH George AE Franssen E Patterns of motor impairment in normal aging, mild cognitive decline, and early Alzheimer’s disease. J Gerontol B Psychol Sci Soc Sci (1997) 52:28–39. 10.1093/geronb/52B.1.P28 9008673

[6] McGough EL Kelly VE Logsdon RG McCurry SM Cochrane BB Engel JM Associations between physical performance and executive function in older adults with mild cognitive impairment: gait speed and the timed “up & go” test. Phys Ther (2011) 91:1198–207. 10.2522/ptj.20100372 21616934 PMC3145896

[7] Aggarwal NT Wilson RS Beck TL Bienias JL Bennett DA. Motor dysfunction in mild cognitive impairment and the risk of incident Alzheimer disease. Arch Neurol (2006) 63:1763–9. 10.1001/archneur.63.12.1763 17172617

[8] McGough EL Cochrane BB Pike KC Logsdon RG McCurry SM Teri L. Dimensions of physical frailty and cognitive function in older adults with amnestic mild cognitive impairment. Ann Phys Rehabil Med (2013) 56:329–41. 10.1016/j.rehab.2013.02.005 23602402 PMC5562508

[9] Fitzpatrick AL Buchanan CK Nahin RL DeKosky ST Atkinson HH Carlson MC Associations of gait speed and other measures of physical function with cognition in a healthy cohort of elderly persons. J Gerontol A Biol Sci Med Sci (2007) 62(11):1244–51. 10.1093/gerona/62.11.1244 18000144

[10] Montero-Odasso M Muir SW Speechley M. Dual-task complexity affects gait in people with mild cognitive impairment: the interplay between gait variability, dual tasking, and risk of falls. Arch Phys Med Rehabil (2012) 93(2):293–9. 10.1016/j.apmr.2011.08.026 22289240

[11] Doi T Makizako H Shimada H Yoshida D Ito K Kato T Brain atrophy and trunk stability during dual-task walking among older adults. J Gerontol A Biol Sci Med Sci (2012) 67(7):790–5. 10.1093/gerona/glr214 22174190

[12] Hooghiemstra AM Ramakers IHGB Sistermans N Pijnenburg YAL Aalten P Hamel REG 4C Study Group. Gait speed and grip strength reflect cognitive impairment and are modestly related to incident cognitive decline in memory clinic patients with subjective cognitive decline and mild cognitive impairment: findings from the 4C Study. J Gerontol A Biol Sci Med Sci (2017) 72(6):846–54. 10.1093/gerona/glx003 28177065

[13] Ng TP Feng L Nyunt MS Feng L Niti M Tan BY Nutritional, physical, cognitive, and combination interventions and frailty reversal among older adults: a randomized controlled trial. Am J Med (2015) 128(11):1225–36. 10.1016/j.amjmed.2015.06.017 26159634

[14] Wolf-Klein GP Silverstone FA. Weight loss in Alzheimer’s disease: an international review of the literature. Int Psychogeriatr (1994) 6(2):135–42. 10.1017/S1041610294001705 7865701

[15] Buchman AS Boyle PA Wilson RS Tang Y Bennett DA Frailty is associated with incident Alzheimer’s disease and cognitive decline in the elderly. Psychosom Med (2007) 69:483–9. 10.1097/psy.0b013e318068de1d 17556640

[16] Yaffe K Barnes D Nevitt M Lui LY Covinsky K A prospective study of physical activity and cognitive decline in elderly women: women who walk. Arch Intern Med (2001) 161(14):1703–8. 10.1001/archinte.161.14.1703 11485502

[17] Kluger A Gianutsos JG Golomb J Wagner A Jr Wagner D Scheurich S Clinical features of MCI: motor changes. Int Psychogeriatr (2008) 20:32–9. 10.1017/S1041610207006461 18072982

[18] Kelaiditi E Cesari M Canevelli M Van Kan GA Ousset PJ Gillette-Guyonnet S Cognitive frailty: rational and definition from an (IANA/IAGG) international consensus group. J Nutr Health Aging (2013) 17(9):726–34. 10.1007/s12603-013-0367-2 24154642 PMC12878905

[19] Verghese J Wang C Lipton RB Holtzer R. Motoric cognitive risk syndrome and the risk of dementia. J Gerontol A Biol Sci Med Sci (2013) 68(4):412–8. 10.1093/gerona/gls191 22987797 PMC3593614

[20] Ng TP Feng L Nyunt MSZ Larbi A Yap KB. Frailty in older persons: multisystem risk factors and the frailty risk index (FRI). J Am Med Dir Assoc (2014) 15(9):635–42. 10.1016/j.jamda.2014.03.008 24746590

[21] Folstein MF Folstein SE McHugh PR “Mini-mental state”: a practical method for grading the cognitive state of patients for the clinician. J Psychiatr Res (1975) 12(3):189–98. 10.1016/0022-3956(75)90026-6 1202204

[22] Nasreddine ZS Phillips NA Bédirian V Charbonneau S Whitehead V Collin I The Montreal Cognitive Assessment, MoCA: a brief screening tool for mild cognitive impairment. J Am Geriatr Soc (2005) 53(4):695–9. 10.1111/j.1532-5415.2005.53221.x 15817019

[23] Ng TP Niti M Chiam PC Kua EH Ethnic differences in cognitive performance on mini-mental state examination in Asians. Am J Geriatr Psychiatry (2007) 15(2):130–9. 10.1097/01.JGP.0000235710.17450.9a 17272733

[24] Ng TP Feng L Lim WS Chng MS Lee TS Tsoi T Montreal Cognitive Assessment for screening mild cognitive impairment: variations in test performance and scores by education in Singapore. Dement Geriatr Cogn Disord (2015) 39:176–85. 10.1159/000368827 25572449

[25] Lezak MD Neuropsychological Assessment. USA: Oxford University Press (2004).

[26] Yeo D Gabriel C Chen C Lee S Loenneker T Wong M Pilot validation of a customized neuropsychological battery in elderly Singaporeans. Neurol J South East Asia (1997) 2:123.

[27] Wechsler D WAIS-III: Wechsler Adult Intelligence Scale Third Edition Administration and Scoring Manual. San Antonio, TX: Psychological Corporation/Harcourt Brace (1997).

[28] Benedict HRB Brief Visuospatial Memory Test–Revised Professional Manual. Odessa, FL: Psychological Assessment Resources, Inc (1997).

[29] Schmidtke K Olbrich S. The clock reading test: validation of an instrument for the diagnosis of dementia and disorders of visuo-spatial cognition. Int Psychogeriatr (2007) 19(2):307–21. 10.1017/S104161020600456X 17147844

[30] Cheung RW Cheung MC Chan AS. Confrontation naming in Chinese patients with left, right or bilateral brain damage. J Int Neuropsychol Soc (2004) 10(1):46–53. 10.1017/S1355617704101069 14751006

[31] Satz P Uchiyama CL White T Color Trail Test: Professional Manual. Odessa: Psychological Assessment Resources (1996).

[32] Feng L Chong MS Lim WS Ng TP. The modified mini-mental state examination test: normative data for Singapore Chinese older adults and its performance in detecting early cognitive impairment. Singapore Med J (2012) 53(7):458–62. 22815014

[33] Portet F Ousset PJ Visser PJ Frisoni GB Nobili F Scheltens P Mild cognitive impairment (MCI) in medical practice: a critical review of the concept and new diagnostic procedure. Report of the MCI working group of the European Consortium on Alzheimer’s disease. J Neurol Neurosurg Psychiatry (2006) 77(6):714–8. 10.1136/jnnp.2005.085332 16549412 PMC2077456

[34] Lee CK Collinson SL Feng L Ng TP. Preliminary normative neuropsychological data for an elderly Chinese population. Clin Neuropsychol (2012) 26(2):321–34. 10.1080/13854046.2011.652180 22288384

[35] O’Bryant SE Waring SC Cullum CM Hall J Lacritz L Massman PJ Staging dementia using clinical dementia rating scale sum of boxes scores: a Texas Alzheimer’s research consortium study. Arch Neurol (2008) 65:1091–5. 10.1001/archneur.65.8.1091 18695059 PMC3409562

[36] Fried LP Tangen CM Walston J Newman AB Hirsch C Gottdiener J Frailty in older adults: evidence for a phenotype. J Gerontol A Biol Sci Med Sci (2001) 56(3):146–57. 10.1093/gerona/56.3.M146 11253156

[37] Ware JE Keller SD Kosinski M SF-12: How to Score the SF-12 Physical and Mental Health Summary Scales. Lincoln, RI: QualityMetric (1998).

[38] Siebeling L Wiebers S Beem L Puhan M ter Riet G Validity and reproducibility of a physical activity questionnaire for elderly. Clin Epidemiol (2012) 4:171–80. 10.2147/CLEP.S30848 22866018 PMC3410686

[39] Tinetti ME Performance-oriented assessment of mobility problems in elderly patients. J Am Geriatr Soc (1986) 34(2):119–26. 10.1111/j.1532-5415.1986.tb05480.x 3944402

[40] Nyunt MSZ Jin AZ Fones CSL Ng TP. Criterion-based validity and reliability of the Geriatric Depression Screening Scale (GDS-15) in a large validation sample of community-living Asian older adults. Aging Ment Health (2009) 13(3):376–82. 10.1080/13607860902861027 19484601

[41] Ng TP Niti M Chiam PC Kua EH. Prevalence and correlates of functional disability in multiethnic elderly Singaporeans. J Am Geriatr Soc (2006) 54:21–9. 10.1111/j.1532-5415.2005.00533.x 16420194

[42] Niti M Chiam PC Kua EH Ng TP. Physical and cognitive domains of the instrumental activities of daily living: validation in a multiethnic population of Asian older adults. J Gerontol A Biol Sci Med Sci (2006) 61(7):726–35. 10.1093/gerona/61.7.726 16870636

[43] Watson NL Rosano C Boudreau RM Simonsick EM Ferrucci L Sutton-Tyrrell K Executive function, memory, and gait speed decline in well-functioning older adults. J Gerontol A Biol Sci Med Sci (2010) 65(10):1093–100. 10.1093/gerona/glq111 20581339 PMC2949334

[44] Feng L Nyunt MS Gao Q Feng L Lee TS Tsoi T Physical frailty, cognitive impairment, and the risk of neurocognitive disorder in the Singapore longitudinal ageing studies. J Gerontol A Biol Sci Med Sci (2017) 72(3):369–75. 10.1093/gerona/glw050 27013397

[45] Stella F Radanovic M Balthazar ML Canineu PR de Souza LC Forlenza OV. Neuropsychiatric symptoms in the prodromal stages of dementia. Curr Opin Psychiatry (2014) 27(3):230–5. 10.1097/YCO.0000000000000050 24613979

[46] Van der Mussele S Mariën P Saerens J Somers N Goeman J De Deyn PP Behavioral syndromes in mild cognitive impairment and Alzheimer’s disease. J Alzheimers Dis (2014) 38(2):319–29. 10.3233/JAD-130596 23963290

[47] Forrester SN Gallo JJ Smith GS Leoutsakos JM Patterns of neuropsychiatric symptoms in mild cognitive impairment and risk of dementia. Am J Geriatr Psychiatry (2016) 24(2):117–25. 10.1016/j.jagp.2015.05.007 26209222 PMC4646727

[48] Heywood R Gao Q Nyunt MSZ Feng L Chong MS Lim WS Hearing loss and risk of mild cognitive impairment and dementia: findings from the Singapore Longitudinal Ageing Study. Dement Geriatr Cogn Disord (2017) 43(5–6):259–68. 10.1159/000464281 28420004

[49] Roberts RO Christianson TJ Kremers WK Mielke MM Machulda MM Vassilaki M Association between olfactory dysfunction and amnestic mild cognitive impairment and Alzheimer disease dementia. JAMA Neurol (2016) 73(1):93–101. 10.1001/jamaneurol.2015.2952 26569387 PMC4710557

[50] Lindbergh CA Dishman RK Miller LS. Functional disability in mild cognitive impairment: a systematic review and meta-analysis. Neuropsychol Rev (2016) 26(2):129–59. 10.1007/s11065-016-9321-5 27393566

[51] Jekel K Damian M Wattmo C Hausner L Bullock R Connelly PJ Mild cognitive impairment and deficits in instrumental activities of daily living: a systematic review. Alzheimers Res Ther (2015) 7(1):17. 10.1186/s13195-015-0099-0 25815063 PMC4374414

[52] Avila-Funes JA Amieva H Barberger-Gateau P Le Goff M Raoux N Ritchie K Cognitive impairment improves the predictive validity of the phenotype of frailty for adverse health outcomes: the three-city study. J Am Geriatr Soc (2009) 57:453–61. 10.1111/j.1532-5415.2008.02136.x 19245415

[53] Samper-Ternent R Al Snih S Raji MA Markides KS Ottenbacher KJ. Relationship between frailty and cognitive decline in older Mexican Americans. J Am Geriatr Soc (2008) 56:1845–52. 10.1111/j.1532-5415.2008.01947.x 18811611 PMC2628807

